# Supplementary material for: Short-term depression and long-term plasticity together tune sensitive range of synaptic plasticity
Source: PLoS Comput Biol. 2020 Sep 25;16(9):e1008265. doi: 10.1371/journal.pcbi.1008265 (PMC7549837; doi:10.1371/journal.pcbi.1008265)
Supplement: Attachment — Submitted filename: plos_comp_biol_reviewer_responses_final.pdf [file pcbi.1008265.s001.pdf]

June 24, 2020

PLOS Computational Biology MS # PCOMPBIOL-D-19-01244

Dear Dr. Hermann Cuntz and Dr. Samuel Gershman,

We would like to resubmit our manuscript "Short-term depression and long-term plasticity together tune sensitive range of synaptic plasticity" (PCOMPBIOL-D-19-01244) for publication in PLOS Computational Biology.

Following the reviewers suggestions, we have substantially extended our investigations and modified the manuscript. In particular, following major comment #2 by Reviewer #1, we have expanded the calcium-based plasticity model to include a coincidence dependent NMDAR component. We now show that the conclusions drawn on the cortical area specificity in terms of firing rate range where synaptic changes are sensitive to spike-timing correlations do not depend on the implementation of the calcium dynamics in the model (see new section starting on pg. 13 and new Fig. 6).

Following major comments #1 and #2 by Reviewer #2, we modified the calcium-based plasticity model in order to account for the coupling between synaptic strength and calcium dynamics. In consequence, all model variants were fitted again to the experimental data, parameter values have been updated, and all the plasticity figures (Figs. 2, 3, 4, 5 and 6) have been revised accordingly. We agree that the coupled model is now in line with the claim of our study, namely investigating the interplay between short- and long-term plasticity dynamics

These additional results from the new analyses strengthen and refine the conclusions of our study. The manuscript has been modified to alleviate all concerns of both reviewers (major modifications in blue), and detailed responses to all their points are included below.

After these extensive changes, we hope the manuscript is now suitable for publication in the PLOS Computational Biology.

We apologize for the long delay of this revision.

Best regards,
Nicolas Deperrois
Michael Graupner

Reviewer # 1

We thank the reviewer for the careful reading of the manuscript and constructive comments. The reviewer's insightful suggestions and concerns helped to improve the model as well as refine our results and the conclusions reached.

Major concerns:

Page 6: Induction of long-term depression is mediated by presynaptic NMDAr and CB1 receptor as a retrograde messenger in somatosensory cortex (Sjostrom et al., 2003). The model does not include these components but rather relies on the postsynaptic NMDAr instead. The arguments are missing if such simplification can be made. Are the conclusions on the spike-timing- and frequency dependence of synaptic plasticity, firing rate ranges of synaptic changes, differences in synaptic plasticity parameters between somatosensory- and visual cortex valid?

We agree with the reviewer that the diversity of long-term plasticity induction and expression mechanisms across different synapses in an important outcome of numerous experimental studies and has to be keep in mind when considering simplified models.

Molecular studies have identified two key elements for the induction of long-term synaptic plasticity in hippocampus and neocortex. First, postsynaptic calcium entry mediated by N-methyl-D-aspartate receptors (NMDARs) (Malenka and Bear 2004) and voltage-dependent Ca^{2+} channels (VDCCs) (Magee and Johnston 2005; Bender et al. 2006; Nevian and Sakmann 2006) has been shown in many cases to be a necessary (Mizuno et al. 2001; Ismailov et al. 2004; Nevian and Sakmann 2006) and sufficient (Malenka et al. 1988; Neveu and Zucker 1996; Yang et al. 1999) signal for the induction of synaptic plasticity. Second, calcium in turn triggers downstream signaling cascades involving protein kinases (mediating LTP) and phosphatases (mediating LTD) (see e.g. Colbran 2004; Wang et al. 2005; O'Connor et al. 2005; Munton et al. 2004). Another G-protein coupled LTD induction pathway involves retrograde signaling by endocannabinoids which requires postsynaptic calcium elevations (Di Marzo et al. 1994; Sjöström et al. 2003; Nevian and Sakmann 2006). Depending on type of synapse, age, and induction protocol different types and combinations of signaling cascades provide the link between the activity-dependent postsynaptic calcium signal and expression mechanisms of synaptic plasticity such as number and/or phosphorylation level of postsynaptic AMPA receptors, or changes in presynaptic transmitter release probability (Malenka and Bear 2004).

Specifically for somatosensory cortex plasticity, Nevian and Sakmann (2006) showed that mGluR activation results in PLC-dependent synthesis of endocannabinoids, which acts as retrograde messengers to induce LTD. Sjöström et al. (2003) showed in visual cortex that activity-dependent postsynaptic release of cannabinoids induces LTD via presynaptic CB1 receptors, which also necessitates the activation of presynaptic NMDA receptors. In both cases, LTD induction requires postsynaptic calcium elevations and postsynaptic calcium buffers abolish LTD (as well as LTP) (Sjöström et al. 2003; Nevian and Sakmann 2006).

The diversity and complexity of the induction pathways is not resolved due to the fact that our model is, by design, highly simplified. The price to pay with this simplification is that it becomes impossible to make the model compatible with all these molecular experiments at different synapses, while the benefit is that it is possible to obtain analytical formulas that allow us to systematically explore parameter space and to fit experimental data.

Specifically, the model retains the postsynaptic calcium signal as a crucial trigger of plasticity. In fact, the behavior of the model in response to spike-timing- and rate dependence during regular and irregular stimulation patterns arises from the interplay between postsynaptic calcium dynamics and the depression and potentiation thresholds which implement in a highly simplified fashion calcium-dependent signaling cascades leading to synaptic potentiation (e.g., kinases) and depression (e.g., phosphatases or G protein-coupled pathways modulated by calcium), respectively. We therefore believe that our findings are general despite the diversity of down-stream induction cascades. How the combination shapes plasticity in particular for irregular stimulation patterns remains to be studied.

We have added a paragraph to the discussion reminding the reader of the biological diversity in plasticity induction and expression mechanisms, and discussing the generality of our results in light of this (pg. 17, lines 605-624).

Page 4:
The joint effect of the pre- and postsynaptic neuronal activity on the calcium concentration is described as the sum of pre- and postsynaptically evoked calcium amplitudes in the differential equation of calcium concentration (Eq 3). However, NMDAr shows a nonlinear behaviour that depends on the postsynaptic membrane potential, thus, on the temporal difference D. How will this nonlinearity affect the results and conclusions made?

Following the reviewer's suggestion, we have extended our investigations to examine a calcium model that accounts for a coincidence dependent NMDAR current (similar to the nonlinear calcium model in the supplement of Graupner and Brunel 2012). Results are summarized in a new section (page 13, "Calcium-based model with nonlinear calcium dynamics") and in Fig. 6. In that model variant ("2. Nonlinear calcium model", see line 138), the calcium transient elicited by a postsynaptic spike consists of two components : (i) the voltage-dependent calcium channel (VDCC) mediated part characterized by C_{post} (second term on the right hand side of Eq. (6)); and (ii) the nonlinear NMDA part controlled by the parameter η (third term on the right hand side of Eq. (6)). η characterizes the increase of the NMDA mediated current in case of coincident presynaptic activation and postsynaptic depolarization. η is related to the experimentally measured nonlinearity factor, n, (Nevian and Sakmann 2006; peak calcium amplitude normalized to the expected linear sum of pre- and postsynaptically evoked calcium transients, see Eq. (7)). We use for the maximal attainable nonlinearity factor $n = 2$ consistent with data from Nevian and Sakmann (2006). The nonlinear implementation follows otherwise the sim-

ple nature of the calcium dynamics model: (i) the nonlinear current exhibits immediate rise time and the same single exponential decay as the linear pre- and postsynaptically evoked calcium currents, (ii) the amount of presynaptic activation at the time of the postsynaptic spike is taken to be proportional to the presynaptically evoked calcium current, *i.e.*, the NMDA-mediated current evoked by the presynaptic spike subjected to short term-depression dynamics and the current synaptic weight (Eq. (3)).

In order to compare the results from the "nonlinear calcium model" with the "linear calcium model", we first fit the nonlinear calcium-based model to the visual- (Sjöström et al. 2001) and somatosensory cortex (Markram 1997) data-sets (note that following a comment by Reviewer #2, all models were refitted since the presynaptically evoked calcium component depends now on the current synaptic weight; see Eq. (3)). The calcium-based model with the nonlinear calcium implementation provides a good fit of the regular spike-pair data-sets (Fig. 6, Tab. 1). We then investigated the STDP curves for regular- and irregular spike-pairs at an intermediate rate (Fig. 6*C,D*), and varied the firing rate for a given Δt and a fixed correlation between pre- and post-spikes to quantitatively compare the impact of firing rate changes and correlations on synaptic changes. Strikingly, despite the fact that the detailed shape of the STDP curve is different in calcium-based model with nonlinear calcium dynamics, the conclusions drawn do not depend on the implementation of the calcium dynamics. Namely :

- When comparing synaptic changes induced by irregular spike-pairs to those induced by regular spike-pairs: (i) For irregular spike-pairs the baseline is elevated compared to regular spike-pairs. (ii) The variation around the baseline synaptic change is reduced for irregular spike-pairs compared to regular spike-pairs (compare Fig. 6*C,D* and Fig. 3). Both differences are further enhanced in the nonlinear calcium model.

- The sensitivity to correlations reaches a peak at low firing rates ($\sim$ 10 spk/sec in visual cortex; 4 spk/s in somatosensory cortex) and vanishes as the firing rates are increased further. The sensitivity to correlations falls in the range of prevalent firing rates in both structures. The specificity in sensitivity to correlations is enhanced in magnitude in the nonlinear calcium model compared to the linear model for the visual cortex data (Fig. 6*G*).

In summary, the extension of the model by a nonlinear version of the calcium dynamics demonstrates that the conclusions drawn on the restriction in sensitivity to spike-timing correlations to cortex specific firing rate ranges in the calcium-based model do not depend on the details of the calcium implementation. As the results do not depend on the calcium implementation, we use the calcium-based model with linear calcium-dynamics in the remainder of the investigations in the manuscript.

Minor concerns:

Page 3:
The term describing the dynamics of the synaptic efficacy in the absence of pre- and

postsynaptic activity is missing in Eq 1. if compared to Graupner and Brunel (2012). Please make it clear why the model is chosen to be multistable, not bistable.

In contrast to the model used in Graupner and Brunel (2012), in the absence of activity, the synapse has a continuum of stable states in Eq. (1). In other words, w is stable at every value (0, 1) for $c < \theta_d, \theta_p$. This modification does not influence the conclusions drawn in this study. In the present study, the mean synaptic weight variable is considered right after the stimulation protocol for the change in synaptic strength. In Graupner and Brunel (2012), a population of individual synapses is simulated, each subjected to synaptic noise and the change in synaptic strength is determined by the fraction of synapses in UP and DOWN states long time after the stimulation protocol. The mean dynamics of the synaptic weight yields very similar results in terms of synaptic change.

We chose here to use a simpler version of the model to draw attention to the main message of the present manuscript, namely how short-term plasticity, firing rate and spike-time correlations shape long-term synaptic changes. In fact, the previously published model (Graupner and Brunel 2012) gets often criticized for its bistable nature, which is still a controversial topic in the field. The results of the present study do not depend on the stability nature of the synapse and we reflected this in the model simplification chosen.

Page 5:
Why and how was the potentiation threshold chosen not to be optimized in the experiments for visual cortex? (Table 1)

Initially, we fixed that threshold to stabilized the fitting routine. The model is highly non-linear due to the thresholds which is why fitting it to experimental data is always a time-consuming process. Fixing the thresholds facilitates the convergence to a global minima in terms of the 'sum of squared distances'.

We agree with the reviewer that this choice is arbitrary and we have therefore included θ_p in the parameters to be optimized by the fitting routine and refitted the entire parameter set under these conditions. See Tab. 1 for the new parameter set including the threshold θ_p obtained through fitting the coupled calcium-based plasticity model to the experimental in visual- and somatosensory cortex.

Page 5:
Should be "The short-term plasticity parameters in the first two lines are obtained from ..." instead of "The short-term plasticity parameters shown in the first two lines are obtain from... " (Table 1)

This was corrected. Thank you for carefully reading the manuscript and pointing out this typo.

Reviewer # 2

We thank the reviewer for the careful reading of the manuscript and constructive comments. The reviewer's insightful suggestions and concerns helped to improve the model as well as refine our results and the conclusions reached.

Major points:

** In the model, short-term plasticity affects postsynaptic calcium changes, which drive long-term synaptic changes, but these synaptic weight changes seem to be decoupled (?) from postsynaptic calcium responses. How would the results change if synaptic weight w affected Cpre (presynaptically evoked calcium amplitude)?*

We agree with that the decoupling between synaptic weight and calcium was a simplification which did not conform with the claim of our study, namely investigating the interplay between short- and long-term plasticity dynamics. We therefore followed the reviewers suggestions and modified the calcium-based plasticity model in order to account for the coupling between synaptic strength and calcium responses. As a first general approach, we assume that the presynaptically induced calcium amplitude C_{pre} scales linearly with the current synaptic strength w (see Eq. (3)). This implementation is equivalent to a modification of the utilized pre-synaptic resources (U, scaled by w; see Eq. (3)) through long-term plasticity changes. The modification adds another dynamical variable, besides activity-dependent changes due to short-term plasticity, to the evoked calcium transients increasing the change in amplitudes upon each presynaptic stimulation.

As a consequence, all model variants - the linear calcium-based model, the linear calcium-based model without STD dynamics, and the nonlinear calcium-based model - were fitted again to the experimental plasticity data obtained in visual- and somatosensory cortex, the parameter values have been updated (Tab. 1) and all plasticity figures have been revised (see Figs. 2, 3, 4, 5 and 6).

Coupling synaptic weight and calcium dynamics resulted in a selective enhancement of LTD for the visual cortex parameter set (compare Fig. 1*A* and 2*A* of this response letter). When LTD was induced, the reduction in synaptic weight and the consequential decrease of the presynaptically induced calcium transient further restricts the calcium concentration to the depression range (above θ_d and below θ_p). This is effect is more pronounced for the visual cortex data featuring a larger presynaptically evoked calcium amplitude C_{pre} (see Tab. 1).

The modification of the model creates effectively a system of coupled, ordinary differential equations between the synaptic weight w and postsynaptic calcium c. In turn, we cannot utilize the diffusion approximation anymore used to calculate analytically the change in synaptic strength (Graupner and Brunel 2012). Rather we moved to an

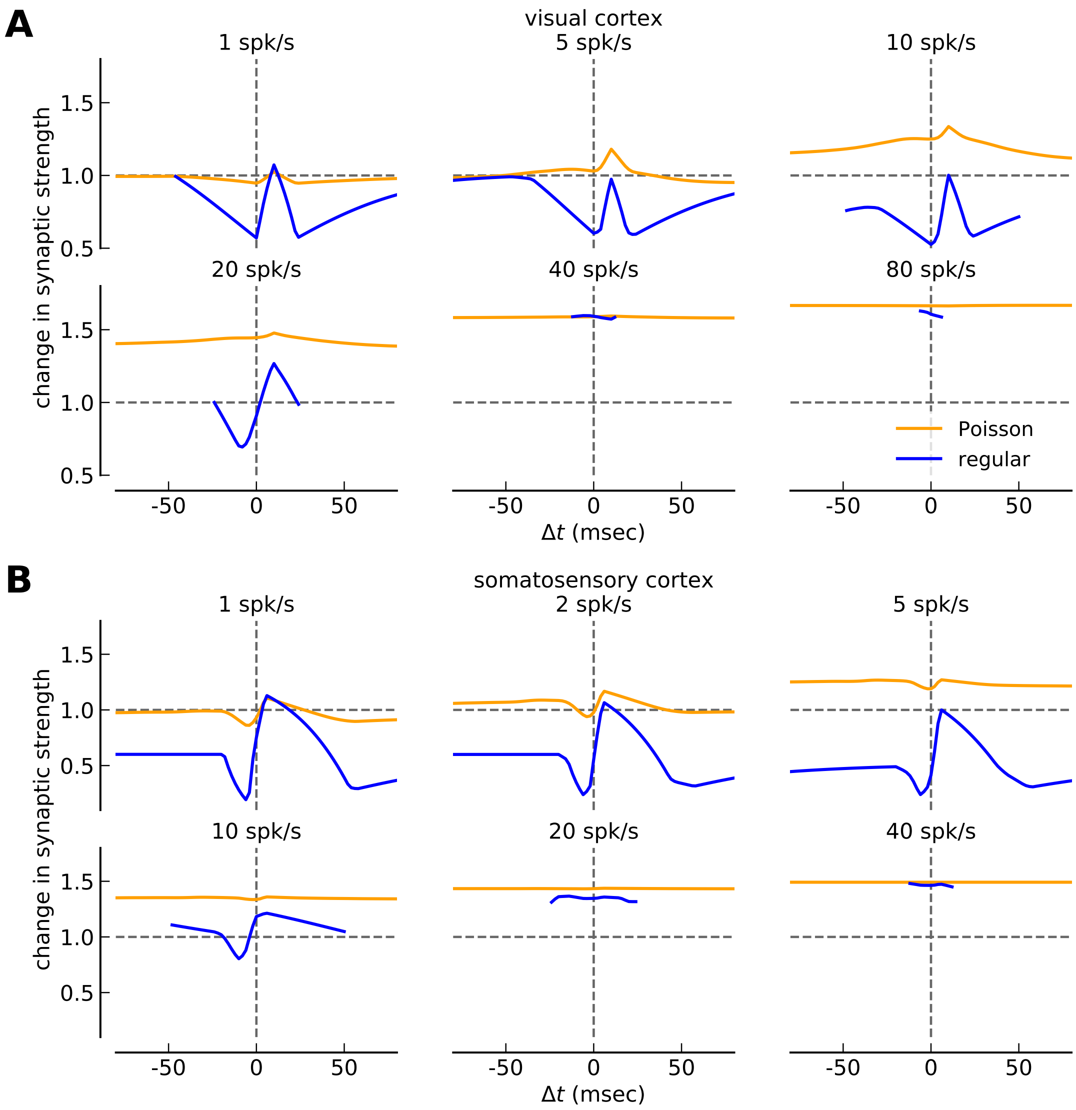

Figure 1: **Change in synaptic strength in response to irregular and regular spike-pairs at different firing rates for the *decoupled* synaptic weight - calcium dynamics model.** ***(A, B)*** See caption of Fig. 3 in the manuscript for the details. Basically, this is the figure which was part of the previous version of the manuscript.

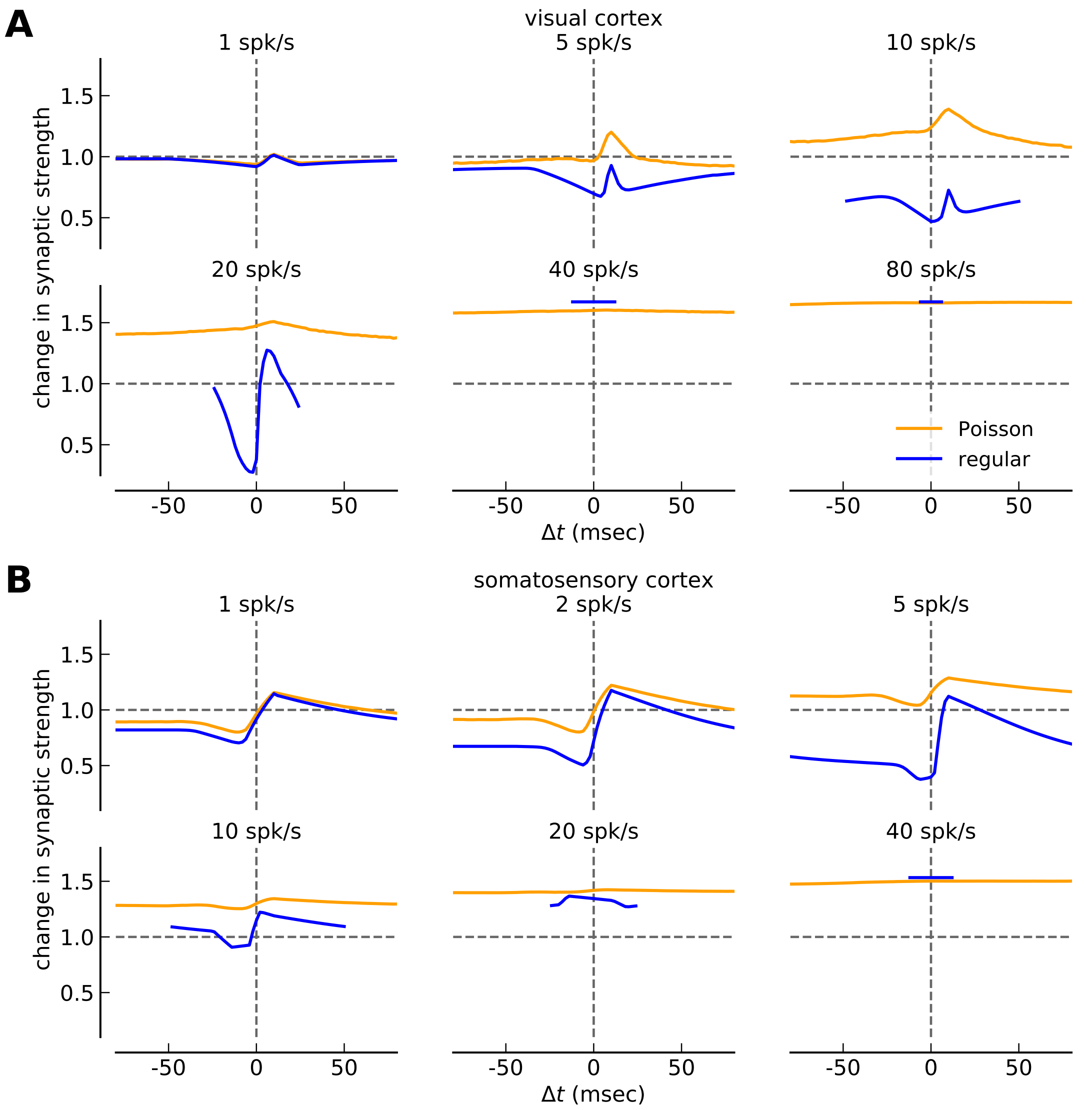

Figure 2: **Change in synaptic strength in response to irregular and regular spike-pairs at different firing rates for *coupled* synaptic weight - calcium dynamics model.** ***(A, B)*** See caption of Fig. 3 in the manuscript for the details. This is the new figure which is part of the revised version of the manuscript.

event-based integration in which calcium and the synaptic efficacy are updated in an analytically exact way upon the occurrence of pre- and postsynaptic spikes. See Higgins et al. (2014) for details of the event-based implementation.

** A related point: The authors argue that due to separation of time scales (line 389), LTD/LTP can be decoupled from short- term depression. Their argument is that LTD/LTP induction protocols last from a few minutes to seconds while the expression of long-term plasticity takes tens of minutes. At least for LTP that does not seem to be the case. The expression of LTP (e.g. in the form of insertion of AMPARs) can be a fast process (e.g. upon high-frequency stimulation)*

We have removed that statement since the model resolves now the coupling between synaptic strength, short-term dynamics and calcium dynamics. See our response to comment #1 of the reviewer.

** When translating voltage events into calcium events, the authors assumed that "the evoked postsynaptic current is directly proportional to the induced calcium concentration". Is this assumption based on spine calcium imaging data or is it a simplified assumption? Please explain in the paper.*

This is an important simplifying assumption of the model. We have moved the paragraph highlighting this assumption from the Methods to the Results section (page 8, lines 251-258). In this paragraph, we explain that STD is characterized in electrophyisological studies in terms of the postsynaptic current or voltage response and that we assume a linear relation to the induced calcium amplitude.

** Has someone tested experimentally the important (previously published) prediction of the calcium-based model that irregular spike-pairs decrease the impact of spike-timing on synaptic changes? The authors should make clear whether this is still yet to be shown in experiments.*

We jumped ahead of ourselves regarding the prediction that irregular spike-pairs reduce the impact of spike-timing on plasticity. We are currently involved in an ongoing collaboration with experimentalists to probe how irregular firing patterns affect STDP. Testing the irregular spike-pair protocol at corticostriatal synapses in slices, we found a good match of the experimental data with this prediction which was obtained from both the calcium- and the triplet-based plasticity models Graupner et al. (2016).

Nevertheless, at this stage the reduction of the impact of spike-timing on synaptic plasticity remains a prediction from Graupner et al. (2016) and we now point that out in the corresponding results section (page 11, lines 387-392).

** Authors write that "dissimilar sensitivities to firing rate changes between visual- and somatosensory cortex are the result of [the] difference in potentiation thresholds." Is there experimental evidence for different plasticity thresholds in these two brain regions or is it a prediction of the model?*

We are not aware of any experimental study aimed at testing plasticity thresholds in the visual- and somatosensory cortex. The difference in potentiation thresholds is a model prediction and we have clearly marked this in the text now (page 13, lines 439-443).

It would be a great test for the model if the predictions on calcium amplitudes and plasticity thresholds (*i.e.* the calcium sensitivity of downstream signaling cascades) can be tested experimentally without perturbing too much spine calcium dynamics and signaling cascades. We always considered the representation of real biophysical variables allowing to make experimentally testable predictions a strength of the model. In Graupner and Brunel (2012), we suggested that differences in the calcium transients can potentially explain the differences observed in plasticity data obtained in hippocampal slices, hippocampal cultures and visual cortex slices.

** Recent papers showed that fast compensatory/homeostatic (non-Hebbian) forms of synaptic plasticity such as fast heterosynaptic plasticity (Zenke et al. Nat Comm 2015) or fast BCM-like metaplasticity (Jedlicka et al. Plos CB 2015) on a time scale of seconds to tens of seconds can account for plasticity data and, importantly, stabilize firing rates and Hebbian plasticity (Zenke & Gerstner, Philos Trans R Soc Lond B Biol Sci. 2017). How could these rapid forms of homeostatic plasticity affect the interaction between short-term plasticity and long-term plasticity? It might be interesting to briefly discuss this in the Discussion.*

We thank the reviewer for reminding us of this important aspect when considering plasticity in the network setting. We actually think that the implementation of short-term plasticity in the calcium-based model acts as a selective compensatory mechanisms for presynaptic activity by effectively implementing an activity-dependent sliding thresholds.

We have added a paragraph to the Discussion on compensatory mechanisms and discuss the model in regard to stabilizing Hebbian plasticity (pg. 16, lines 563-578).

-

Minor points:

Figure 1 legend: the text needs to distinguish between data and simulations. E.g. in Fig. 1B the authors could replace "Example calcium traces" e.g. by "Example simulations for calcium traces". Fig.1A –are data points from somatosensory cortex missing? Based on what data was STD in the somatosensory cortex fitted?

We agree with the reviewer that the transition from experimental data, model fitted to data and simulated data was not clearly marked in the caption of Fig. 1. We have completely rewritten the caption of panel *(A)* and point out at the end of the figure caption that all calcium dynamics simulations in that figure (*B-E*) are generated using the calcium-based plasticity model with STD dynamics (Eqs. (2), (3)).

Somatosensory short-term depression has been described and fitted using the same STD

model in (Loebel 2009). We use the STD parameters proposed in that study. For our comparative study, we use the same STD model and fit it to EPSP amplitudes obtained in the visual cortex. We hope that the rewritten caption of Fig. 1 and the paragraph in lines 259-268 in the results section further highlight this approach.

Figure 4: To emphasize the differences in firing rate-dependency of synaptic plasticity between visual and somatosensory cortex, it would make sense to use the same scale (0 – 80 spk/s) for the x-axis.

Initially, we produced this figure according to the reviewers suggestion. However, the details of the firing rate and correlation induced changes in plasticity in somatosensory cortex occurring up to 10 spk/s cannot be perceived in that depiction. We therefore opted for the different x-axis scales.

The difference in the x-axis ranges is pointed out the caption of Fig. 4. We furthermore added a mention of this difference in the text (page 13, line 410). We think that the point of different prevalent firing rates and sensitivity ranges comes across since the two columns of panels in Fig. 4 show a strong qualitative resemblance because they are plotted on very different firing rate ranges. In other words, sensitivity of plasticity to firing rate changes and correlations exhibit similar shapes but occur at very different firing rates.

Line 61: "To changes in synaptic efficacy under more natural conditions, we use irregular spike patterns" should read "To simulate changes in..."

We added the missing word.

Line 315: "at time lag delta-t which probability p." should read "at time lag delta-t with probability p.

Corrected.

Text for Table 1: In the sentence "The short-term plasticity parameters shown in the first two lines are obtain from fitting the STD model to", "obtain" should read "obtained"

Thanks. We corrected this error.

Fig. 4: The correlation variable p is not explained in the legend. Add the explanation. (See Fig 5 legend, in which it is explained).

Good point, we added the definition in the results text (page 11, lines 376-379) and in the caption of Fig. 4.

Line 422: replace "reduced" by "reduce"

Thank you for carefully reading the manuscript and pointing out this and the other typos above.

References

Bender, V. A., K. J. Bender, D. J. Brasier, and D. E. Feldman (2006, April). Two coincidence detectors for spike timing-dependent plasticity in somatosensory cortex. *The Journal of Neuroscience: The Official Journal of the Society for Neuroscience 26*(16), 4166–4177.

Colbran, R. J. (2004, September). Protein phosphatases and calcium/calmodulin-dependent protein kinase II-dependent synaptic plasticity. *The Journal of neuroscience : the official journal of the Society for Neuroscience 24*(39), 8404–9.

Di Marzo, V., A. Fontana, H. Cadas, S. Schinelli, G. Cimino, J. C. Schwartz, and D. Piomelli (1994, December). Formation and inactivation of endogenous cannabinoid anandamide in central neurons. *Nature 372*(6507), 686–691.

Graupner, M. and N. Brunel (2012, February). Calcium-based plasticity model explains sensitivity of synaptic changes to spike pattern, rate, and dendritic location. *Proceedings of the National Academy of Sciences of the United States of America 109*(10), 3991–3996.

Graupner, M., P. Wallisch, and S. Ostojic (2016, November). Natural Firing Patterns Imply Low Sensitivity of Synaptic Plasticity to Spike Timing Compared with Firing Rate. *Journal of Neuroscience 36*(44), 11238–11258.

Higgins, D., M. Graupner, and N. Brunel (2014, October). Memory maintenance in synapses with calcium-based plasticity in the presence of background activity. *PLoS computational biology 10*(10), e1003834.

Ismailov, I., D. Kalikulov, T. Inoue, and M. J. Friedlander (2004, November). The kinetic profile of intracellular calcium predicts long-term potentiation and long-term depression. *The Journal of Neuroscience: The Official Journal of the Society for Neuroscience 24*(44), 9847–9861.

Loebel, A. (2009). Multiquantal release underlies the distribution of synaptic efficacies in the neocortex. *Frontiers in Computational Neuroscience 3*(November), 27.

Magee, J. C. and D. Johnston (2005, June). Plasticity of dendritic function. *Current Opinion in Neurobiology 15*(3), 334–342.

Malenka, R. C. and M. F. Bear (2004, September). LTP and LTD: An embarrassment of riches. *Neuron 44*(1), 5–21.

Malenka, R. C., J. A. Kauer, R. S. Zucker, and R. A. Nicoll (1988, October). Postsynaptic calcium is sufficient for potentiation of hippocampal synaptic transmission. *Science (New York, N.Y.) 242*(4875), 81–84.

Markram, H. (1997, January). Regulation of Synaptic Efficacy by Coincidence of Postsynaptic APs and EPSPs. *Science 275*(5297), 213–215.

Mizuno, T., I. Kanazawa, and M. Sakurai (2001, August). Differential induction of LTP and LTD is not determined solely by instantaneous calcium concentration: An essential involvement of a temporal factor. *The European journal of neuroscience 14*(4), 701–8.

Munton, R. P., S. Vizi, and I. M. Mansuy (2004, June). The role of protein phosphatase-1 in the modulation of synaptic and structural plasticity. *FEBS letters 567*(1), 121–8.

Neveu, D. and R. S. Zucker (1996, May). Long-lasting potentiation and depression without presynaptic activity. *Journal of Neurophysiology 75*(5), 2157–2160.

Nevian, T. and B. Sakmann (2006, October). Spine Ca2+ signaling in spike-timing-dependent plasticity. *The Journal of neuroscience : the official journal of the Society for Neuroscience 26*(43), 11001–13.

O'Connor, D. H., G. M. Wittenberg, and S. S.-H. Wang (2005). Dissection of Bidirectional Synaptic Plasticity Into Saturable Unidirectional Processes. *Journal of Neurophysiology 94*(2), 1565–1573.

Sjöström, P. J., G. G. Turrigiano, and S. B. Nelson (2001, December). Rate, timing, and cooperativity jointly determine cortical synaptic plasticity. *Neuron 32*(6), 1149–1164.

Sjöström, P. J., G. G. Turrigiano, and S. B. Nelson (2003, August). Neocortical LTD via coincident activation of presynaptic NMDA and cannabinoid receptors. *Neuron 39*(4), 641–654.

Wang, X., T. Lu, R. K. Snider, and L. Liang (2005, May). Sustained firing in auditory cortex evoked by preferred stimuli. *Nature 435*(7040), 341–6.

Yang, S. N., Y. G. Tang, and R. S. Zucker (1999, February). Selective induction of LTP and LTD by postsynaptic [Ca2+]i elevation. *Journal of neurophysiology 81*(2), 781–7.
